# The experiences and perceptions of wellbeing provision among English ambulance services staff: a multi-method qualitative study

**DOI:** 10.1186/s12913-022-08729-1

**Published:** 2022-11-15

**Authors:** Viet-Hai Phung, Kristy Sanderson, Gary Pritchard, Fiona Bell, Kelly Hird, Paresh Wankhade, Zahid Asghar, Niro Siriwardena

**Affiliations:** 1 Community and Health Research Unit, School of Health and Social Care, University of Lincoln, Lincoln, United Kingdom; 2 School of Health Sciences, University of East Anglia, Norwich, United Kingdom; 3 Yorkshire Ambulance Service NHS Trust, Wakefield, United Kingdom; 4 Edge Hill University Business School, Edge Hill University, Ormskirk, United Kingdom

**Keywords:** Mental health and wellbeing, Ambulance, Emergency medical services

## Abstract

**Background:**

NHS ambulance service staff are at risk of poor physical and mental wellbeing because of the likelihood of encountering stressful and traumatic incidents. While reducing sickness absence and improving wellbeing support to ambulance staff is a key NHS priority, few studies have empirically documented a national picture to inform policy and service re-design. The study aimed to understand how ambulance service trusts in England deal with staff health and wellbeing, as well as how the staff perceive and use wellbeing services.

**Methods:**

To achieve our aim, we undertook semi-structured telephone interviews with health and wellbeing leads and patient-facing ambulance staff, as well as undertaking documentary analysis of ambulance trust policies on wellbeing. The study was conducted both before and during the UK first COVID-19 pandemic wave. The University of Lincoln ethics committee and the Health Research Authority (HRA) granted ethical approval. Overall, we analysed 57 staff wellbeing policy documents across all Trusts. Additionally, we interviewed a Health and Wellbeing Lead in eight Trusts as well as 25 ambulance and control room staff across three Trusts.

**Results:**

The study highlighted clear variations between organisational and individual actions to support wellbeing across Trust policies. Wellbeing leads acknowledged real ‘tensions’ between individual and organisational responsibility for wellbeing. Behaviour changes around diet and exercise were perceived to have a positive effect on the overall mental health of their workforce. Wellbeing leads generally agreed that mental health was given primacy over other wellbeing initiatives. Variable experiences of health and wellbeing support were partly contingent on the levels of management support, impacted by organisational culture and service delivery challenges for staff.

**Conclusion:**

Ambulance service work can impact upon physical and mental health, which necessitates effective support for staff mental health and wellbeing. Increasing the knowledge of line managers around the availability of services could improve engagement.

**Supplementary Information:**

The online version contains supplementary material available at 10.1186/s12913-022-08729-1.

## Introduction

Emergency responders may encounter extreme critical incidents, including trauma and violence [1, 2]. Some of their work has been characterised as dangerous [3, 4]. Shift working may impact many aspects of the personal and professional lives of ambulance staff, including their physical and psychological wellbeing [5, 6].

In England, NHS ambulance services provide emergency medical services (EMS). The nature of ambulance work means that staff are more likely to be exposed to traumatic incidents. These traumatic incidents can exact a toll on their mental health and wellbeing. Moreover, other factors, such as workload, shift work, limited time for debriefing, and lack of recognition can aggravate staff mental health and wellbeing [7].

Ambulance service respondents to the 2018 NHS staff survey indicated more discrimination, less equal opportunities, and higher work-related stress. This resulted in illness and poorer employee engagement than other NHS organisations [8]. These problems were aggravated by a national paramedic shortage and high turnover rates [9]. In a recent study, Asghar et al. (2021) demonstrated that sickness rates for clinical ambulance staff varied considerably over time and by ambulance trust in England [10].

Ambulance staff are at risk of poor wellbeing with high rates of Post-Traumatic Stress Disorder (PTSD), anxiety and depression [7, 11, 12]. They may also have a greater risk of occupational suicide than the general population [13, 14]. In a UK survey by the charity Mind of over 1,300 ambulance service respondents, problems at work were often cited as the main cause of mental health problems. These included: excessive workload; pressure from management; long hours; changing shift patterns; and exposure to distressing or traumatic incidents [15].

The pandemic has raised concerns about its psychological impact on frontline healthcare professionals [16]. Ambulance staff are the first response to seriously unwell and injured patients, including those symptomatic with COVID-19. In 2021, a Mind survey identified that almost one-third of UK ambulance staff respondents described their mental health as poor or very poor, with 77 per cent stating their mental health had deteriorated during the COVID pandemic [17].

Unfortunately, research into staff sickness absence among ambulance staff suggests limited evidence on the effectiveness of interventions to reduce absence [18]. A recently published evidence map of mental health, wellbeing and support interventions for UK ambulance services staff noted a scarcity of published data on whether such interventions were effective [19]. Research showed that ambulance staff feel unsupported by employers when suffering work-related poor wellbeing and welfare services provision [7].

Ambulance staff face difficulties in staying well at work, but the effectiveness of wellbeing support is not well understood. Furthermore, it is not clear how these offerings are developed and implemented, nor how ambulance staff understand, access, and value the resources provided by their employer or from external agencies. For the purposes of this paper wellbeing support is defined as information and programmes ambulance services have in place to promote staff mental health and wellbeing, which may include direct provision of health and wellbeing programmes, signposting, or referral including to clinical services such as occupational health.

We aimed to examine the knowledge, experience, and perceptions of ambulance and control room staff in England about employer and informal provision of mental health and wellbeing interventions. In addition, we examined variations in English ambulance service policies and strategies for staff wellbeing. The study focused on specific mental well-being strategies, as well as suicide prevention strategies and interventions.

We aimed to address the following research questions:

- To what extent and how do ambulance trusts address mental health and wellbeing in their policies?
- How do Health and Wellbeing Leads perceive these policies, service provision, and delivery?
- How do ambulance and control room staff perceive and experience mental health and wellbeing service provision?

## Methods

The study was planned before the SARS-CoV2 pandemic and conducted both before and during the UK first pandemic wave. We undertook a documentary analysis, interviews with wellbeing Leads as well as ambulance and control room staff. These are described in further depth below.

### Design and setting

The first element of the multi-method study was a directed-content documentary analysis of English NHS ambulance service Trusts (denoted A to J) staff wellbeing policies (Appendix 1). These were supplemented by semi-structured telephone interviews with wellbeing lead informant interviews exploring policy implementation (Appendix 2); and with ambulance and control room staff who interacted with patients either face-to-face or on the telephone from three NHS ambulance trusts in England (C, I, and G) (Appendix 3). These trusts represented services with high, medium or low relative sickness absence rates [10].

### Documentary analysis

Policy documents related to mental health and wellbeing were requested and collated from each Trust. This included stand-alone staff wellbeing policies, in addition to other policies that could include health-related actions (such as return to work policy, break policy). In the directed content analysis, each document was reviewed for the presence of any recommended action related to health. Each action was coded as either Primary, Secondary or Tertiary prevention. We also categorised whether the intervention was targeted at Individuals (PI, SI and TI) or Organisations (PO, SO and TO) [20]. Primary actions identify or manage risk and protective factors for health and wellbeing (HWB). These actions focus on support and guidance on healthy lifestyle change as protective factors for good mental health and wellbeing.

Secondary actions are those that support employees at risk of developing poor HWB. There is a stress risk assessment to identify people at risk and potentially in need of intervention. Finally, tertiary actions support individuals who are already affected by poor HWB. Tertiary actions were exemplified by return to work conversations by line manager. Individual actions include healthy lifestyle action and participating in any HWB opportunities. Those targeted at the organisational level include policies defining specific roles, responsibility and job design. We summarise findings for the stand-alone wellbeing policies, then all other policies.

### Interviews with health and well-being leads and patient-facing ambulance staff

The documentary analysis was supplemented with semi-structured telephone interviews with eight out of the ten ambulance Trust wellbeing leads in England. Although our participants had different job titles, we refer to them all as “wellbeing leads” as they contributed to designing and delivering staff wellbeing support and to preserve anonymity. Wellbeing leads were predominately from a health professional or human resources/organisational development background.

#### Recruitment

We also undertook semi-structured telephone interviews with frontline ambulance and control room staff in participating NHS Trusts [21–23] (Appendix 2–3). They were invited to contact the research team to express their interest in being interviewed through a variety of indirect internal communications routes, including newsletters and social media.

#### Ethics

All participants were given a participant information sheets explaining the purpose of the research. They also received consent forms that outlined what their involvement would entail. Participants could withdraw from the study at any time and did not have to justify doing so. Participants who withdrew, could request that any non-anonymised data is erased. Following anonymisation of the data, it was not possible to withdraw any data. Prior to participation, we acquired informed consent from all participants.

All research was conducted in accordance with the principles of informed consent (Table 1). The University of Lincoln’s Health & Social Care Ethics Committee granted ethical approval (ref 2019-Aug-0723) and the study received Health Research Authority (HRA) approval (ref IRAS ID 261,350, REC reference 19/HRA/3658).

**Table 1:** Principles of ethical research

1	Research staff and subjects must be fully informed about purpose, methods and intended possible uses of research, what participation entails, and what risks, if any, involved
2	Confidentiality of information supplied by research subjects and anonymity of respondents must be respected
3	Research participants must participate voluntarily, free from coercion
4	Harm to research participants must be avoided
5	Independence and impartiality of researchers must be clear, and any conflicts of interest or partiality must be explicit

Source: Silverman, D. (2010) “Ethical research” in Silverman, D. (ed.) Doing qualitative research—third edition. London: Sage Publications Ltd

#### Data collection and analysis

We digitally recorded each staff interview to better capture the dialogue. The interviews were transcribed verbatim. Staff telephone interviews continued until data saturation, where further interviews produced “*no new data, no new themes, no new coding and ability to replicate the study*” [24].

Once the interview transcripts were returned, they were coded and analysed using Framework Analysis (FA) [25]. The initial stage of FA was familiarisation through reading transcripts, supplemented by the hand-written notes during the interview. The themes were coded and analysed using NVivo 12 [26]. The next stage was to establish the main themes [26, 27]. FA offers flexibility by retaining the a priori codes and supplementing them with de novo codes [25, 26]. Indexing organised and categorised the data into the appropriate themes, categories, and codes within the established main thematic framework. After indexing, we charted the data into the relevant headings and sub-headings. Following charting, we mapped and interpreted the interview data. The main headings and verbatim quotes from the interview data are set out in Appendix 4.

## Results

### Documentary analysis

Eight out of ten English ambulance trusts provided an overarching staff wellbeing policy. Trusts A and J were unable to provide their policy as it was under revision. Table 2 presents a breakdown of the directed-content analysis of the primary staff wellbeing policy documents. The total number of actions in the primary staff wellbeing policy per action type is presented. The percentages show the distribution of total actions across action type. For example, Trust I’s policy had 299 total actions, with nearly three-quarters of these actions classified as tertiary (72.9 per cent). This compares with Trust C where only 4.2 per cent of actions were classified as tertiary. There was more emphasis on primary preventative actions (72.5 per cent) (Table 1).

**Table 2:** Directed-content analysis of primary staff wellbeing policies (not including Trust A and J)

Trust	PI action numbers (percentage)	PO action numbers (percentage)	SI action numbers (percentage)	SO action numbers (percentage)	TI action numbers (percentage)	TO action numbers (percentage)	TOTAL number of actions
Trust C	14 (11.7)	73 (60.8)	8 (6.7)	20 (16.7)	0 (0)	5 (4.2)	120
Trust I	4 (1.3)	17 (5.7)	7 (2.3)	53 (17.7)	57 (19.1)	161 (53.8)	299
Trust F	11 (5.2)	171 (80.3)	2 (0.9)	25 (11.7)	0 (0)	4 (1.9)	213
Trust E	5 (7.9)	52 (94.6)	1 (1.6)	5 (7.9)	0 (0.0)	0 (0.0)	63
Trust D	0 (0.0)	45 (84.9)	0 (0.0)	6 (11.3)	0 (0.0)	2 (3.8)	53
Trust B	9 (5.9)	24 (15.8)	12 (7.9)	60 (39.5)	8 (5.3)	39 (25.7)	152
Trust G	9 (5.3)	125 (73.1)	1 (0.6)	27 (15.8)	3 (1.8)	6 (3.5)	171
Trust H	0 (0.0)	79 (98.8)	0 (0.0)	1 (1.3)	0 (0.0)	0 (0.0)	80
Trust J	50 (5.3)	365 (38.9)	110 (11.7)	404 (43.0)	2 (0.2)	9 (0.9)	939
Trust A	14	73	8	20	0	5	120

*PI* Primary Individual, *PO* Primary Organisational, *SI* Secondary Individual, *SO* Secondary Organisational, *TI* Tertiary Individual, *TO* Tertiary Organisational

**Fig. 1:**
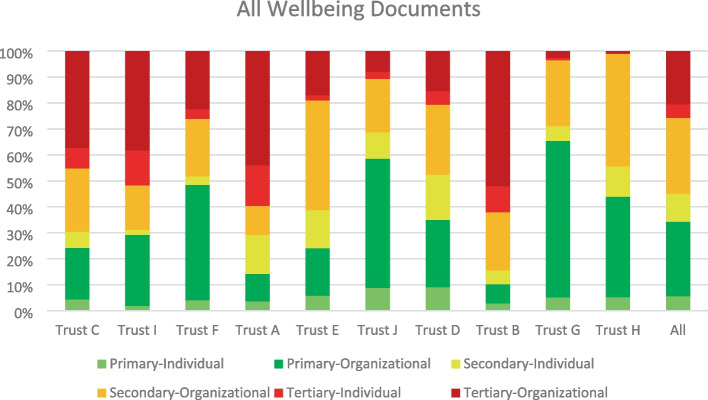
Distribution of action types in content of all policy documents for all trusts

All ten trusts provided a variety of other staff wellbeing policy documents (e.g., absenteeism management; drug, alcohol and substance misuse; meal break; post-incident care; domestic abuse). A total of 57 documents were analysed across Trusts. Figure 1 shows the distribution of content by action type for all wellbeing policy documents reviewed.

### Informant interviews

**Fig. 2:**
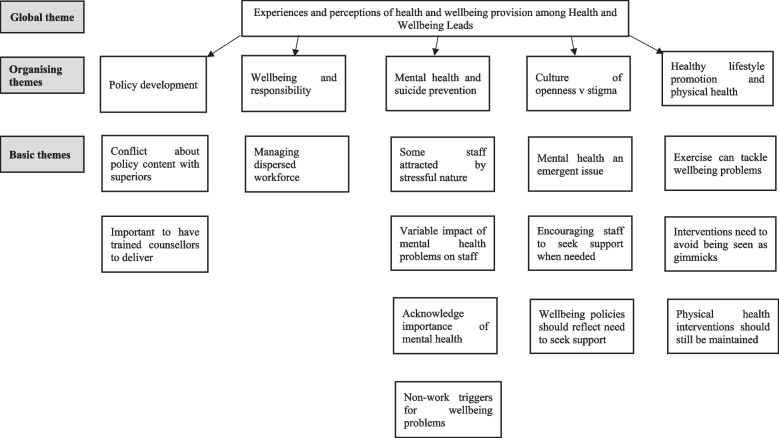
Thematic map for interviews with health and wellbeing leads

We undertook semi-structured telephone interviews with a Health and Wellbeing Lead in eight ambulance trusts in England. The themes from the interviews are outlined below in Fig. 2 and Appendix 2.

### Individual staff interviews

**Fig. 3:**
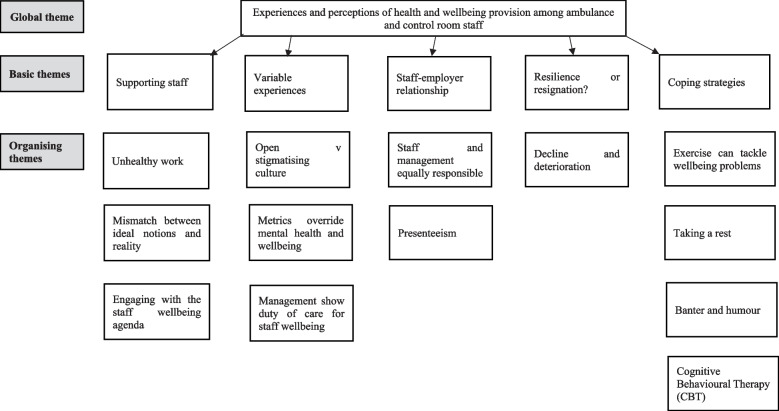
Thematic map for ambulance and control room staff interviews

We undertook 25 semi-structured telephone interviews with ambulance staff from Trust C (16), Trust I (2) and Trust G (7) from April-November 2020. From these interviews, the main themes were: supporting staff; engagement with staff wellbeing; variable experiences of health and wellbeing; resilience or resignation; and coping strategies for stress and ill-health (Fig. 3 and Appendix 3).

### Key themes

The need to support staff with their mental health and wellbeing is an ongoing concern for ambulance trusts.

#### Policy development

All decisions required senior management approval within each trust. Trust H explained after a long negotiation, the trust had the final right of veto: *“a number of recommendations, two of which are about to go to trust for approval*” (Trust H). Decisions around what is included in, and omitted from, wellbeing policy sometimes led to conflict between wellbeing leads and their superiors. In the following quote, the wellbeing professional describes their long-term struggle to establish what they regarded as an essential addition to the trust’s welfare provision:

‘*I fought for three long and hard years, with management, high up, and I mean the board, everybody, to put in place my own recruited, trained, bespoke counselling team who were experienced and trained to deal with trauma.*’ (Trust F).

#### Unhealthy work

The nature of ambulance work can be stressful because ambulance staff can often encounter distressing incidents, experience violence or aggression. Long hours doing physically, and mentally draining work take their toll on ambulance staff. The ageing process means that everyone physically and mentally declines as they get older. For ambulance staff, the physical and mental decline is likely to be more pronounced given the rigours of the job.

‘*And they kind of sort of sat me down and gave me a tissue because I was crying, and he told me that I should have more confidence. And that was basically the end of it*.’ (Staff 3, Trust G).

‘*We work such awful hours. There are loads of studies that show our life expectancy and our health is reduced over the long term because of the type of work we do*.’ (Staff 2, Trust C).

‘*Now I’m getting older and getting a bit creakier…I needed to get myself fit in several ways – general stamina, but also upper body strength*.’ (Staff 16, Trust C).

Telephone interactions with patients or the general public can be just as stressful as those undertaken face-to-face. Interviewees raised concerns about the risks associated with ambulance work to both their physical and mental health during the COVID-19 pandemic.

‘*Well, I think the Police take more than us, but the ambulance service, we do take a lot of crazy stuff. But we’re only human. We do still get personally affected by it*.’ (Staff 6, Trust G).

‘*I got assaulted at work about five weeks ago and the lack of support I’ve had from the Trust*….’ Staff 3, Trust C.

‘*Yes. You know, “You’re crap, you need a new job.” You think, “Hang on a minute, you’ve called for help, and all you’ve done is shout at me from the moment I picked the phone up*.’ (Staff 1, Trust I).

#### Open v stigmatising organisational cultures

The wellbeing lead at Trust J speculated that there has been a cultural shift around stigma and mental health. This cultural shift has increased demand for mental health and wellbeing support as people are, in these circumstances, more likely to seek help. The need for openness and willingness to disclose in order to access support was reflected in staff wellbeing policies.

‘*Culturally, it is more acceptable to come forward with mental health issue, and I think we’ve had to try and embed that internally as well, which has been particularly challenging within the ambulance service, because when I joined 13 years ago, it was very much ‘stiff upper lip and carry on*.’ (Trust J).

‘*It is … imperative that we continue to foster an open culture of disclosure to encourage staff to seek the right support when they need it*.’ (Trust E policy).

Culture can influence how organisations deal with mental health and wellbeing. Interviewees experienced a range of organisational cultures on the open-closed continuum. Experiences of trying to get support from health and wellbeing services within their ambulance Trust, and overall, the experience of services were variable. Staff found it easier to deal with mental health and wellbeing problems if there was an open culture. Such a culture was one where colleagues could discuss problems with others and managers proactively concerning themselves about staff health and wellbeing.

‘*Oh, definitely, yes. I don’t know. There is just a happier vibe. We have got the director of the 111 service in the [region Trust G]. He comes round every weekend and he says hello to every member of staff*.’ (Staff 4, Trust G).

‘*My manager, particularly, is so lovely. He is so helpful. I’ve got a few health problems. Whenever I’ve got a problem, I have to take some time off. He’s the first person to come and ask me if I’m okay and have a chat with me about what was wrong and*, “*What are you doing to*…? *Have you spoken to someone*?”’ (Staff 5, Trust G).

‘*I mean I’ve had colleagues before, like when I’ve been with supervisors when they’ve had very difficult- this is just an example, this isn’t- you know, hangings or* [something has 0:16:05] *really affected somebody*. *They’ve let them go home*, *they’ve said*, “*You need to go home*.” *They’re very good at taking control and saying*, “*This is actually for your mental health, not anybody else’s*.” “*I mean you’re saying that you’re okay, but I can see that you’re not. You need to go home*.”’ (Staff 6, Trust G).

‘*Even the… I don’t want to say the second most important person, but the second highest in command out of the whole service came in and had a chat with us and put everyone’s mind at rest. He learned all of our names. He said that he personally… If anyone needed to have a chat, if anyone was worried about anything, we could contact him. It was so lovely that someone so high up, with so much responsibility and so many other things to worry about, was so caring and so considerate to the way we were feeling. That was just really, really, lovely, so I thought I’d mention that*.’ (Staff 5, Trust G).

By contrast to these open cultures, stigmatising organisational cultures existed where immediate managers were perceived to lack empathy and support. Such cultures exist where employees are not encouraged to speak up about their personal health and wellbeing concerns.

‘*There is an understanding there. But, with a private company who has just been giving a contract saying*, “*Do this service*.” *You have got to have something about you, and they didn’t. It was atrocious*.’ (Staff 4, Trust G).

In such a culture, some staff felt compelled to work, even if not fully able to do so (presenteeism) under pressure from management because they wanted to help the public. Some interviewees appeared to perceive a lack of recognition from their managers about the mental and physical toll the job takes on ambulance staff. This perceived lack of support was seen as causing some staff to work despite problems remaining unresolved. It also highlighted a perceived inconsistency in approach to absence management and support.

‘*Yes. The support, from management, is nowhere near as good as the support you get outside. They, very much, just want you to be doing your job. They don’t want you to be off work. They just want you to be there doing it. They don’t want to be paying you if you’re not there, things like that. The other support you get is brilliant. Even with management… I think it happens most places, doesn’t it, management just sort of want you to man up a bit, get on with it, if that’s the right thing to- Well I did get told that once by management, to be honest*, “*Just man up*.” *That didn’t go down very well*.’ (Staff 1, Trust G).

‘*It’s taken all my will power not to go in this weekend….I’m on antibiotics, now, for an infection…..it’s not anything major but I- I wanted to go in this weekend because I know they’re going to be busy and it’s my team, but I had to think of myself. I’m really only back to normal the last 6 months, after 18 months, so I can’t afford- As much as I want to go in, I can’t afford to risk it really at the minute*.’ (Staff 1, Trust C).

‘*The ones* [employees] *they can rely on; they don’t seem to value them. The ones that they think*, “*Oh yes, she’ll turn up whatever happens*.”’ (Staff 1, Trust C).

While continuing to work through illness can bring benefits to some people [28], on balance, participants described a presenteeism that could be considered coercive and potentially harmful. There was also guilt around staff self-isolating during COVID-19 when they may be needed.

‘*You're sitting at home feeling a bit of a fraud really, because I'm getting paid to sit at home, you know*?’ (Staff 8. Trust I).

For some interviewees, their experiences of mental health and wellbeing support did not always match their preconceived notions of what should happen. The apparent emphasis of management on metrics and adopting a business-like model, seemed inconsistent with a duty of care to staff, who also perceived a lack of empathy. Some felt these metrics were prioritised at the expense of staff health and wellbeing.

‘*Yes, and it goes against the vision and values that they put in front of you and make you preach at your interview that it’s about patient care and getting best treatment at the best time. Even on jobs you’ve got them on your back*, “*How long you’re going to be? You’ve been on this job whatever time*.” *It’s like*, “*I’m waiting to speak to a doctor*.” *I’m the one that’s seen the patient. I’m the one that’s going to be stood up in front of the court*.’ (Staff 2, Trust G).

#### Variable experiences of health and wellbeing in the workplace

Organisational culture influenced the nature of ambulance staff experiences of mental health and wellbeing in the workplace. While there were those who had bad experiences of support from mental health and wellbeing services in their ambulance trust, the picture was nuanced in that there were others who had better experiences, in this example of counselling. However, COVID-19 impacted the availability of service provision.

‘*Yes. When I was at an all-time low and struggling, they left me two weeks, so they’re not going to be bothered about someone that’s just worried because of their job*.’ (Staff 2, Trust G).

‘*They always listen to you…We’re quite familiar with each other now, and you build up a bit of a relationship with them. When you need to access things, it’s always there. They’ll always be someone, in one of the services, who will answer the phone or get back to you as quick as they can*.’ (Staff 1, Trust G).

‘*When I was going through my divorce, I worked for a different service then. They were supportive and they put in obviously referrals to see a counsellor, but my GP also put in a service as well. It came through at the same time, but I just used the Ambulance Service one because obviously it was better using the work one. I found that quite helpful and got a lot of things off my chest that I suppose you don’t realise bothers you until you start talking about things*.’ (Staff 2, Trust G).

‘*I know the NHS service is absolutely inundated. I've just been discharged unfortunately because of the coronavirus and they're not doing face-to-face. The woman that I was seeing was leaving, so they've discharged me without completing the course. Given me some online stuff to do*.’ (Staff 4. Trust I).

Many participants discussed Trauma Risk Management (TRiM), which is peer-delivered and designed to be an ongoing support system that helps individuals manage traumatic events. This intervention needs to be separated from counselling ser/vices. However, after an initial assessment, individual employees can be referred for it. It was described as having efficacy for some staff but was not universally effective. Challenges with the appropriateness of and accessibility of TRiM referrals were raised.

‘*They’ve got the TRiM where if you feel that you need to talk to somebody about a certain call, they’ll listen to the call and be able to give you their opinion and give you some sort of counselling towards if it was a particularly bad one*.’ (Staff 1).

‘*Then they can come back and say, “I need TRiM.” It seems to work for them. However, for others, they go off for months because of it. Not because of TRiM but because it’s not worked*’ (Staff 1, Trust I).

#### Mutual responsibility

The nature of their work puts ambulance staff at increased risk of physical and mental health and wellbeing problems. Thus, the employer is responsible for creating mechanisms and structures that not only respond to the consequences of such occurrences, but also proactively mitigate their impact. Dealing with mental health and wellbeing in the workplace depends on both staff and the organisation taking it seriously. In some cases, immediate line managers and others did; less so in other cases.

‘*I personally think that people do need to take a lot of responsibility for their own wellbeing*.’ Staff 3, Trust C.

‘*They expect you to say*, “*Oh yes, I’ll go in*.” *The ones that go off all the while, it seems to me that they get rewarded. They get all the support they need. One particular guy, since he passed his assessment, he’s not done a straight 6- He’s been off, I would say… He’s been a year, 14 months, I would say he’s probably been off 8 months. Admittedly, his brother… He had a death in the family with his brother, so that knocked him back again. He’d been off before that, previously, so… He’s been getting all the support. You think, “That lady there, that’s been sat there for the last 12 years, that really needs some help and…” They just expect it of the ones that turn up day in and day out*.’ Staff 1, Trust C.

In examining accountability and responsibility, wellbeing leads recognised limits to wellbeing policies in the ambulance service and noted external factors beyond their remit. Just as ambulance staff face obstacles in achieving positive wellbeing [7], so do wellbeing policies and leads. Each ambulance trust has wellbeing teams serving a large number of staff in diverse and mobile or dispersed environments. The Trust H lead described the practical difficulty in meeting the needs of their workforce due to geographical spread.

‘*We cover 6,000 square miles. We’ve got over 65 ambulance stations, as well as a number of other venues that we use…Just being able to communicate with the workforce being so dispersed is the biggest challenge that I think we have. Letting them know what we’re doing, what our plans our, how they can get involved. I think that’s for me the biggest challenge*.’ (Trust H).

The interviews illustrated the reality of how sickness and absence policies, as set out in the documentary analysis, impact upon ambulance staff at ground level with staff struggling to remain in work or feeling supported to recover.

‘*They helped me with juggling some hours, start times and… I’ve said that I think I can’t do 12-hour nights or I can’t do 6:00 to 18:00, 18:00 to 06:00 anymore. They said*, “*Come up with what you want to do, then we’ll see if we can fit it in*?” *I don’t like to think about dropping it, but I think, for myself, I’ve got to because I’m just not sleeping. If can get home by 4:00 o’clock in the morning- They’ve said they’ll look at it*.’ (Staff 1, Trust C).

‘*I’ve also got a colleague that’s been fighting just to reduce her hours for a few hours. She’s been fighting for it for three years. It’s just tipped her over. She lost her husband. As soon as she got back to work, they expected her to be back at work and normal. It just didn’t happen. She’s had no support, really, whatsoever. She’s now off sick*.’ (Staff 1, Trust I).

Lifestyle schemes were generally thought of as positive, and it was noted that schemes like these perform an important symbolic function in these organisations, working almost as a gesture of goodwill.

‘*It makes you feel like, mentally, the Trust is trying to give you back something so you’re getting something out of the Trust rather than them just constantly draining the life out of you*.’ (Staff 2, Trust I).

However, not all wellbeing leads supported these intervention policies if they were not part of a holistic approach to tackling challenges to staff mental health and wellbeing. One initiative from Trust F encouraged staff to use an exercise bike during their breaks and were rewarded with a healthy fruit smoothie if they achieved a set distance. The wellbeing lead viewed these types of interventions as gimmicks that failed to tackle the root causes.

‘*That’s not patronising at all, is it? No, not at all…..I love yoga and Tai Chi, but the idea that things like this will prevent someone from developing chronic stress disorder, or suicidal ideation, or, actually, taking their own lives or depression that is intense and impactful. No, it won’t. None of that* [expletive] *will work*.’ (Trust F).

#### Mental health and suicide prevention

The stressful nature of ambulance work can impact on staff mental health and wellbeing. Wellbeing leads keenly understand that their workers face many role-related pressures or events that may impact upon their mental health, but that there are also people who are attracted to ambulance service by such work.

‘*Of course, those jobs, as horrendous and emotionally demanding as they are, many of our staff, the larger majority of our staff, frankly, that’s why they joined. […] but it gives them a buzz, as opposed to going to the rubbish jobs*.’ (Trust F).

Wellbeing leads described how in recent years, their work around mental health had increased significantly. The centrality of mental health in wellbeing documents seems to only be increasing over time and reflects the diversity of mental health challenges. Having appropriately trained counsellors to work with ambulance staff was considered important to address their mental health and wellbeing.

‘*It’s probably worth noting that we’re putting a lot of effort into our Mental Health framework going forward…I think, anecdotally, mental health is probably a big one at the moment. That can be low-level mental health. It’s not just like for PTSD or addiction forms of mental health. It could be just day-to-day stress and anxiety*.’ (Trust E).

‘*We’re absolutely putting a huge amount of focus into mental wellbeing for our staff and our managers. And it does feel like that is absolutely the priority in terms of where our energy and time is being spent*.’ (Trust H).

They recognise that personal experiences of these stressors will vary, and the Trust has to accommodate diverse needs. Interviewees also understood the pervasive impact of poor mental health for some individuals.

‘*Depression is definitely a big thing, chronic stress, stress that slowly drips and accumulates, which then impacts communication, which impacts how they talk to managers, how they respond to members of the public, how they respond at home*.’ (Trust F).

Many of the most significant mental health related issues addressed through wellbeing services were for non-work-related concerns. Trust B provided the example of employees who experienced trauma through their previous employment. Trust H lead noted that non-work factors may constitute a majority of reasons for seeking formal support.

‘*We've got lots of ex-Forces that end up working for us who've got really significant PTSD from their experiences on tour and have spoken quite openly about historic dependence on things like alcohol. I think it's that self-medicating that people find comforting*.’ (Trust B).

‘*We also see a lot of people with mental-health-related difficulty due to reasons outside of work. Our employee assistance programme that we commission sees two thirds more people accessing that service with personal-related problems than they are with work-related problems*.’ (Trust H).

There are documented cases of ambulance staff suicides and there is a clear sense that trust interventions can reduce instances of suicide, and Trusts are mobilising to identify preventative actions. Despite the widespread understanding that death by suicide and suicidal ideation is an issue within the ambulance service, the policy documents paid little attention to it. Only four Trusts mentioned it at all, while none provided specific guidance. This omission seems even more glaring when considering the working experience of Trust C where staff presenting with suicide ideation is described as a daily occurrence.

#### Coping strategies for stress and ill-health

Most wellbeing lead interviewees understood that behaviour changes around diet, exercise, and movement could improve the overall mental health of their workforce.

‘*Linking mental health with physical exercise, awesome. All these things are brilliant because they make sense. As a human being, if you’re chronically stressed, if you step outside and put one foot in front of the other, you are regaining control of your life*.’ (Trust F).

Physical health concerns were noted, particularly musculoskeletal conditions and injuries, with support for different aspects of health varying over the working life course.

‘*We also do have a high level of people that have musculoskeletal conditions and injuries related to the role. Our absence level for that is pretty close to mental health, to be honest*.’ (Trust H).

‘*Our people at the moment will have to work until they’re 67. So it was interesting to see how sickness levels or people’s needs changed over that period of time in their career*.’ (Trust I).

In response to physical and mental health challenges, other coping strategies included: exercise; banter; black humour; and rest.

‘*I find that a nice release to go and exercise*.’ (Staff 3, Trust C).

‘*I have a five-minute kip and I am right as rain afterwards*.’ (Staff 14, Trust C).

‘*You crack a joke and it doesn't always work, so you try something else to alleviate the situation. But yes, humour is definitely a coping mechanism, without a shadow of a doubt, yes. Definitely*.’ (Staff 2, Trust I).

Staff also used particular tools to tackle mental health and wellbeing issues. Some perceived Cognitive Behavioural Therapy (CBT) to be superior to counselling, but others did not.

‘*I think the CBT has helped me more so, because that gives you the tools […] rather than - Counselling doesn't really give you that*.’ (Staff 4, Trust C).

## Discussion

### Summary of key findings

Despite suicide and suicidal ideation being an issue within the ambulance service, the policy documents paid little attention to it. There is now less stigma associated with mental health, which has facilitated more open discussion around the subject in ambulance services. Consequently, more staff have felt encouraged to seek support for their mental health.

Wellbeing leads recognised limits to wellbeing policies in the ambulance service. These were mainly attributed to external factors beyond their remit. The nature of ambulance work can trigger problems for staff, which may result in them needing to access support for their mental health and wellbeing. HWB leads recognised this, adding also that the nature of this work is what often initially attracts staff to do the work.

Ambulance staff recognised the risks that the nature of their work posed to their mental health and wellbeing. Staff felt that it was the responsibility of management to mitigate these risks. The first step towards this would be to create an open organisational culture where such matters can be discussed. After that, they felt that there should be bespoke interventions that were easily accessible to staff. In response to the challenges to their mental health and wellbeing, staff coped using a variety of strategies, including exercise, CBT, and humour.

### Links to research evidence

Ambulance staff are likely to encounter some very stressful situations [1, 2, 7, 29, 30] which can exact a toll on their physical and mental health [31].

The influence of culture and organisation suggests that changes to both are key to improving staff mental health and wellbeing [32]. However, previous studies examining cultural change in the ambulance services identified managerial ineffectiveness as key factor in the lack of change [33, 34]. In an open organisational culture, colleagues are free to discuss concerns about mental health and wellbeing with each other. Both wellbeing leads and staff interviewees agreed that the ambulance sector had a more open culture regarding mental health. This change in perception is consistent with other studies [17], and may, therefore, improve staff experiences of disclosure [35] of poor wellbeing. Changing perceptions is often the first step to access any support [36].

In this study, the significant effect of management on an individual’s wellbeing at work was described. This effect influenced staff perceptions of the reality of disclosing needs and accessing support both positively and negatively.

While some staff reported feeling valued at work, they were also pressurised to perform by managers. This conflicts with organisational values. Given the lack of training for managers in early recognition of mental health problems [13], it is unsurprising that some staff interviewees felt that managers in ambulance trusts seem unprepared or unresponsive to the challenges of supporting staff. These staff presented with depression, trauma, were subjected to abuse or violence at work or required support for non-work-related problems. This is despite the wellbeing leads and literature recognising the high prevalence of these issues in the ambulance workforce.

Timely access to appropriate services, for example, TRiM or bereavement and divorce support can be challenging. Accessibility for staff is variable. Furthermore, the absence of guidance or policy documents describing financial wellbeing and managing sleep or fatigue represents an opportunity to improve service provision for staff.

Managers are familiar with attendance management processes as shown by universal presence of absence policies, but staff report inconsistent approaches by managers. Less well-recognised is that working through illness can bring psychological, financial and organisational benefits [28]. In this setting, staff identified that ambulance work was associated with physical decline, which should be included in managing attendance at work. An important part of early intervention is managing presenteeism.

However, such a strategy fails to recognise how presenteeism can affect individuals’ ability to deliver their role effectively [37]. Managers are not specifically supported in understanding these nuances of presenteeism, and this is a workforce development opportunity for the ambulance sector and other health and social care settings.

The impact of poor staff wellbeing may be overlooked if the organisational priority is responding to medical emergencies, i.e., through reducing breaks. Focusing on response time targets also neglects other important aspects of patient experience, such as reassurance and interpersonal skills [38] and may also detract from the professionalisation of ambulance staff [31].

There are challenges in delivering a wellbeing programme for ambulance staff. These include: the availability of appropriately trained counsellors; interacting with a large and geographically dispersed workforce; and the tension between organisational and individual responsibility for wellbeing. Also apparent through the documentary analysis is the absence of explicit support for staff with a disability. This presents an opportunity for more emphasis on reducing stigma or discrimination and improving empowerment for attendance and reasonable adjustment.

COVID-19 has created additional challenges for ambulance staff, in their professional and personal lives, including: social distancing; increased risk of infection from patients; and the cumulative increase in health system and service pressures [39]. Not only has COVID-19 impacted significantly on the health and wellbeing of ambulance staff, it will continue to challenge how ambulance services support them [40, 41].

The documentary analysis, informant and staff interviews provided an important sequential approach to, not only mental health and wellbeing provision in ambulance services, but also how they have been delivered and received by staff. In doing so, it provides an understanding of regional variations in provision across ambulance services, their effectiveness and how they could be improved in future. There is potentially an opportunity for shared learning with other emergency services, who face similar demands and stresses. For instance, Oscar Kilo provide staff support services for Police staff, such as wellbeing at work, psychological trauma and risk management, and peer support for wellbeing [42].

While the issues raised are extremely important, they must be seen through the prism of the interviewees being self-selecting. They were keen to talk about their experiences and perceptions of health and wellbeing. Their keenness to participate in, and give their time for, the interviews can suggest that they were already opinionated on health and wellbeing.

### Implications for future research, policy and practice

The study findings raise significant issues with potential implications for how to improve support structures and mechanisms, as well as organisational culture within ambulance trusts, for dealing with health and wellbeing concerns. Further research could examine how other ambulance trusts respond to staff mental health and wellbeing concerns, as well as how ambulance staff experience these services. There is potential for sharing best practice between ambulance trusts because the responses from participants in this study show that there are key elements in an effective response to mental health and wellbeing concerns among their staff.

Firstly, line managers can take a more proactive approach to mental health and wellbeing. A more proactive approach can be more consistently incorporated in staff wellbeing policies across trusts, where there was variation in promotion of proactive versus reactive strategies. In this respect, managers need more training to recognise early signs of challenges to the mental health and wellbeing of their staff [13]. The availability of mental health and wellbeing support services should be advertised widely to staff, with clarity over points of contact and confidentiality to reduce the perceived impact of disclosure. A balance of actions across personal versus organisational responsibility ensures that individuals can exert agency in addressing wellbeing concerns, while receiving the support of an organisation working towards a healthier workplace.

Secondly, there is a case for mental health and wellbeing to be more bespoke to staff needs, including for non-work issues. Currently, some services offer a certain number of counselling sessions, and in some cases, accessibility is inconsistent. Uneven accessibility may lead to situations where staff with ongoing mental health and wellbeing concerns do not have further access to support services.

Thirdly, trusts who respond well to staff concerns are those that recognise the emotional toll of some incidents and allow time for reflection. It must be recognised that the provision of incident management systems is not universally effective. Therefore, a broad range of different services may be required. Fourthly, staff who are absent through physical and/or mental health problems should not feel pressured to return to work before they are ready.

## Conclusions

Ambulance service work can impact upon physical and mental health, which necessitates effective support for staff mental health and wellbeing. Line managers should be trained to identify and implement support for their staff. The effectiveness of the support depends also, partly, on the nature of the organisational culture, which is primarily set by those managers that staff interact directly with alongside the availability, range of and accessibility of any offers.

## Supplementary Information

**Additional file 1:** **Appendix 1.** Content Analysis Trust Breakdown.

**Additional file 2:** **Appendix 2.** Interview Topic Guide.

**Additional file 3:** **Appendix 3.** Interview schedule for telephone interviews with ambulance staff.

Additional file 4: **Appendix 4. **BMC HSR paper themes and quotes.

## Data Availability

The anonymised datasets used and/or analysed during the current study are available from the corresponding author on reasonable request.

## Abbreviations

CBT: Cognitive Behavioural Therapy
HWB: Health and Wellbeing
NHS: National Health Service
PI: Primary- Individual
PO: Primary- Organisational
PTSD: Post-Traumatic Stress Disorder
SI: Secondary- Individual
SO: Secondary- Organisational
TI: Tertiary- Individual
TO: Tertiary- Organisational
TRiM: Trauma Risk Management
