# Supplementary material for: The experiences and perceptions of wellbeing provision among English ambulance services staff: a multi-method qualitative study
Source: BMC Health Serv Res. 2022 Nov 15;22:1352. doi: 10.1186/s12913-022-08729-1 (PMC9664049; doi:10.1186/s12913-022-08729-1)
Supplement: Supplementary file 1 — Additional file 1: Appendix 1. Content Analysis Trust Breakdown. [file 12913_2022_8729_MOESM1_ESM.docx]

# Appendix 1: Content Analysis Trust Breakdown

**Ambulance Trust A**

| **ID** | **TRUST A DOCUMENT** | **PI** | **PO** | **SI** | **SO** | **TI** | **SO** | **Total** |
| --- | --- | --- | --- | --- | --- | --- | --- | --- |
| 1.1. | Occupational Health and Wellbeing Policy | 14 | 73 | 8 | 20 | 0 | 5 | 120 |
| 1.2. | Return to Work Review Meeting Flow Chart | 0 | 0 | 0 | 0 | 0 | 17 | 17 |
| 1.3. | Sickness Absence Management Policy | 8 | 26 | 23 | 102 | 39 | 164 | 362 |
|  |  | **22** | **99** | **31** | **122** | **39** | **186** | **499** |

**Ambulance Trust B**

| **ID** | **TRUST B DOCUMENT** | **PI** | **PO** | **SI** | **SO** | **TI** | **SO** | **Total** |
| --- | --- | --- | --- | --- | --- | --- | --- | --- |
| 2.1. | Attendance and Wellbeing Policy and Procedure | 4 | 17 | 7 | 53 | 57 | 161 | 299 |
| 2.2. | Meal Break Proposal | 4 | 50 | 1 | 8 | 0 | 0 | 63 |
| 2.3 | People Strategy and OD Plan, Objective 6 (draft) | 0 | 45 | 0 | 1 | 0 | 0 | 46 |
| 2.4. | Trauma Risk Management (TRiM) | 0 | 3 | 0 | 10 | 0 | 0 | 13 |
|  |  | **8** | **115** | **8** | **72** | **57** | **161** | **421** |

**Ambulance Trust C**

| **I.D** | **TRUST C DOCUMENTS** | **PI** | **PO** | **SI** | **SO** | **TI** | **SO** | **Total** |
| --- | --- | --- | --- | --- | --- | --- | --- | --- |
| 3.1. | Managing Attendance Policy | 4 | 12 | 11 | 66 | 21 | 119 | 233 |
| 3.2. | Resilience Building Group Work for APPs and IROs | 0 | 10 | 0 | 0 | 0 | 0 | 10 |
| 3.3. | Stress Management and Wellbeing Policy | 8 | 59 | 5 | 35 | 0 | 4 | 111 |
| 3.4. | Wellbeing Strategy | 11 | 171 | 2 | 25 | 0 | 4 | 213 |
|  |  | **23** | **252** | **18** | **126** | **21** | **127** | **567** |

**Ambulance Trust D**

| 4 | **TRUST D DOCUMENTS** | **PI** | **PO** | **SI** | **SO** | **TI** | **SO** | **Total** |
| --- | --- | --- | --- | --- | --- | --- | --- | --- |
| 4.2. | Management of Stress Procedure | 4 | 26 | 6 | 14 | 0 | 0 | 50 |
| 4.4. | Sickness Absence Policy | 8 | 12 | 48 | 26 | 56 | 158 | 308 |
|  |  | **12** | **38** | **54** | **40** | **56** | **158** | **358** |

**Ambulance Trust E**

| 5 | **TRUST E DOCUMENTS** | **PI** | **PO** | **SI** | **SO** | **TI** | **SO** | **Total** |
| --- | --- | --- | --- | --- | --- | --- | --- | --- |
| 5.3. | Dignity at Work Policy and Procedure | 4 | 30 | 23 | 89 | 0 | 0 | 146 |
| 5.4. | Domestic Abuse Guidance | 0 | 5 | 0 | 0 | 5 | 49 | 59 |
| 5.5. | Drug, Alcohol and Substance Misuse Policy | 28 | 28 | 22 | 50 | 1 | 18 | 147 |
| 5.8. | Insulin Dependent Diabetes and Driving | 1 | 11 | 13 | 55 | 0 | 0 | 80 |
| 5.9. | Invest in Yourself Health & Wellbeing Localised Approach Framework Guidance | 5 | 52 | 1 | 5 | 0 | 0 | 63 |
| 5.10. | Policy on Transgender Staff | 0 | 1 | 37 | 89 | 0 | 0 | 127 |
| 5.12. | Sickness Absence Procedure | 4 | 17 | 26 | 80 | 14 | 99 | 240 |
| 5.13. | Smoke Free Policy | 14 | 25 | 7 | 3 | 0 | 0 | 49 |
| 5.14. | The Early Identification and Workplace Support for Specific Learning Difficulties Procedure | 1 | 10 | 14 | 43 | 0 | 0 | 68 |
|  |  | **57** | **179** | **143** | **414** | **20** | **166** | **979** |

**Ambulance Trust F**

| 6 | **TRUST F DOCUMENTS** | **PI** | **PO** | **SI** | **SO** | **TI** | **SO** | **Total** |
| --- | --- | --- | --- | --- | --- | --- | --- | --- |
| 6.1 | Alcohol drugs and work policy Y (HSP) | 17 | 40 | 4 | 14 | 6 | 9 | **90** |
| 6.44 | Stress policy | 2 | 67 | 18 | 30 | 0 | 8 | 125 |
|  |  | **19** | **107** | **22** | **44** | **6** | **17** | **215** |

**Ambulance Trust G**

| **7** | **TRUST G DOCUMENTS** | **PI** | **PO** | **SI** | **SO** | **TI** | **SO** | **Total** |
| --- | --- | --- | --- | --- | --- | --- | --- | --- |
| 7.4 | Depression and Low Mood: A Self-Help Guide to Depression (leaflet) | 0 | 0 | 26 | 0 | 21 | 1 | 48 |
| 7.5 | Desk Posture (leaflet) | 35 | 0 | 12 | 0 | 0 | 0 | 47 |
| 7.6 | Low Back Pain (Leaflet) | 43 | 1 | 12 | 0 | 0 | 0 | 56 |
| 7.7 | Managing Health and Attendance Policy and Procedure | 5 | 43 | 31 | 40 | 39 | 157 | 315 |
| 7.8 | Meal Break Policy | 11 | 46 | 1 | 1 | 0 | 0 | 59 |
| 7.9 | Retirement Policy | 12 | 59 | 32 | 70 | 0 | 0 | 173 |
| 7.10. | Stress: A Self-Help Guide to Stress and Stress Management (leaflet) | 1 | 0 | 32 | 0 | 0 | 1 | 34 |
| 7.11 | Trauma: A Self-Help Guide to Understanding Trauma (leaflet) | 0 | 0 | 51 | 0 | 0 | 1 | 52 |
| 7.12 | Trauma Risk Management (TRiM) Guidance | 0 | 53 | 4 | 132 | 0 | 18 | 207 |
| 7.14 | Wellbeing Hub Managers Guide | 4 | 28 | 1 | 4 | 0 | 2 | 39 |
| 7.15 | Wellbeing Strategy 2017 - 2022 | 0 | 45 | 0 | 6 | 0 | 2 | 53 |
| 7.17 | Your Guide to Wellbeing in XXXXX (leaflet) | 0 | 42 | 11 | 77 | 3 | 6 | 139 |
|  |  | **111** | **317** | **213** | **330** | **63** | **188** | **1222** |

**Ambulance Trust H**

| **8** | **TRUST H DOCUMENTS** | **PI** | **PO** | **SI** | **SO** | **TI** | **SO** | **Total** |
| --- | --- | --- | --- | --- | --- | --- | --- | --- |
| 8.1. | Health and Wellbeing Policy | 9 | 24 | 12 | 60 | 8 | 39 | 152 |
| 8.2. | Health and Wellbeing Toolkit. A Guide to the Three Stages and Associated Meetings | 0 | 0 | 0 | 0 | 5 | 69 | 74 |
| 8.3. | Health and Wellbeing Toolkit. Appeals Procedure | 0 | 0 | 0 | 0 | 6 | 8 | 14 |
| 8.4. | Health and Wellbeing Toolkit. Application Temporary Redeployment | 0 | 0 | 0 | 0 | 3 | 0 | 3 |
| 8.5. | Health and Wellbeing Toolkit. Formal Meetings | 0 | 0 | 0 | 0 | 5 | 29 | 34 |
| 8.6. | Health and Wellbeing Toolkit. Key Principles | 1 | 2 | 7 | 21 | 5 | 6 | 42 |
| 8.7. | Health and Wellbeing Toolkit. Life Changing Life Limiting/Critical Long-Term Conditions | 0 | 0 | 0 | 0 | 4 | 31 | 35 |
| 8.8. | Health and Wellbeing Toolkit. Patterns of Sickness Absence | 0 | 1 | 0 | 0 | 0 | 6 | 7 |
|  |  | **10** | **27** | **19** | **81** | **36** | **188** | **361** |

**Ambulance Trust I**

| **9** | **TRUST I DOCUMENTS** | **PI** | **PO** | **SI** | **SO** | **TI** | **SO** | **Total** |
| --- | --- | --- | --- | --- | --- | --- | --- | --- |
| 9.1. | Health and Wellbeing Strategy* | 9 | 125 | 1 | 27 | 3 | 6 | 171 |
| 9.2. | Stress Policy and Procedure | 7 | 65 | 17 | 53 | 0 | 2 | 144 |
|  |  | **16** | **190** | **18** | **80** | **3** | **8** | **315** |

**Ambulance Trust J**

| **10** | **TRUST J DOCUMENTS** | **PI** | **PO** | **SI** | **SO** | **TI** | **SO** | **Total** |
| --- | --- | --- | --- | --- | --- | --- | --- | --- |
| 10.1. | Attendance at Work Policy | 0 | 17 | 0 | 9 | 0 | 5 | 31 |
| 10.2. | Debrief Guidance | 0 | 0 | 16 | 137 | 0 | 0 | 153 |
| 10.3. | Employee Wellbeing Strategy 2015-20 | 9 | 79 | 2 | 14 | 0 | 3 | 107 |
| 10.4. | Employee Wellbeing Supporting Staff involved in an incident, Complaint or Claim Policy | 3 | 38 | 17 | 102 | 0 | 0 | 160 |
| 10.5. | Employment Break Policy | 0 | 12 | 0 | 0 | 0 | 0 | 12 |
| 10.6. | Fitness for Work Policy | 14 | 51 | 31 | 40 | 2 | 0 | 138 |
| 10.7. | Health and Wellbeing Policy | 0 | 79 | 0 | 1 | 0 | 0 | 80 |
| 10.8. | Issue Resolution (Grievance) Policy | 0 | 5 | 16 | 17 | 0 | 0 | 38 |
| 10.9. | Meal Break Standard Operating Procedure | 16 | 8 | 0 | 2 | 0 | 0 | 26 |
| 10.10. | Post Incident Care Guidance | 0 | 3 | 17 | 45 | 0 | 0 | 65 |
| 10.11. | Working Time Policy | 8 | 73 | 11 | 37 | 0 | 0 | 129 |
|  |  | **50** | **365** | **110** | **404** | **2** | **8** | **939** |
