# Supplementary material for: The experiences and perceptions of wellbeing provision among English ambulance services staff: a multi-method qualitative study
Source: BMC Health Serv Res. 2022 Nov 15;22:1352. doi: 10.1186/s12913-022-08729-1 (PMC9664049; doi:10.1186/s12913-022-08729-1)
Supplement: Supplementary file 2 — Additional file 2: Appendix 2. Interview Topic Guide. [file 12913_2022_8729_MOESM2_ESM.docx]

# Appendix 2: Interview Topic Guide

**1. Background**

Can you describe your tenure at the trust and how you go this this point in your career?

**2. The importance of Health and Wellbeing (HWB) for ambulance sector employees**

What are the main challenges of promoting HWB in the ambulance sector?

What is the most common HWB issue you deal with?

What is the most significant HWB issue you deal with?

**4. Consultation and development process**

What are your HWB policies informed by? How was it developed? Who with?

Can you describe the consultation process that went into the development of your HWB?

**5. Perspectives on Trust’s HWB policies**

What parts of your HWB strategy are working best?

Do you think there are any gaps in your HWB policies?

How has the HWB culture changed since you’ve worked for the trust?

Are there any parts of your HWB strategy need improvement?

Are there any parts of your HWB policy that you are planning to change?

Can you provide an example of a successful intervention?

**<<After section 4 go through the specific information we have sent them about how we have interpreted and coded their policies.**

**6. Implementation strategy**

*****For the trusts with an implementation plan. For those that do, this question will be tailored to them.

How are your HWB policies implemented?

To what extent are these living documents?

**7. Monitoring and outcome**

How are your HWB strategies monitored? [PROMPT: board level’ HR]

What are the KPIs?

**8. Closing question**

Thank you for your time. Before we finish, is there anything you would like to add on the subject of HWB, that we have not already covered?
