# Supplementary material for: The experiences and perceptions of wellbeing provision among English ambulance services staff: a multi-method qualitative study
Source: BMC Health Serv Res. 2022 Nov 15;22:1352. doi: 10.1186/s12913-022-08729-1 (PMC9664049; doi:10.1186/s12913-022-08729-1)
Supplement: Supplementary file 3 — Additional file 3: Appendix 3. Interview schedule for telephone interviews with ambulance staff. [file 12913_2022_8729_MOESM3_ESM.docx]

# Appendix 3: Interview schedule for telephone interviews with ambulance staff

We are looking to find out about your experience of getting help with your mental wellbeing. We are interested in what sort of help is available through your work – we call these employee wellbeing services, and also what you know about that you can access outside of work – we call these non-employee wellbeing services. We want to know about your experience of accessing these, what worked well and why think that was the case, and what wasn’t good for you, and any reasons that you might not have sought these kinds of help. We want to understand all this so that we can suggest improvements.

If you feel uncomfortable at any time then you can ask us to stop, and you can find some help numbers on the PIL.

We will talk through three broad topics, I might ask some extra questions if I want to find out a little more.

**What challenges, if any, to your mental well-being have you faced?**

- Prompt: describe job role.
- Prompt: what types of incidents have you attended to?
- Prompt: to what extent, if any, have the incidents that you have attended had an impact upon your mental well-being?
- Prompt: how have you been affected by the current Covid-19 Pandemic? What coping strategies have you adopted?

**Tell us about the services provided by your employer….**

- Which mental wellbeing services are you aware of in the ambulance service where you work?
- What prompted you to seek that support?
- How easy was it to access the support?
- What problems, if any, did you encounter when trying to access the support?
- Describe what support you were offered.
- How was the support delivered?
- What were you hoping/expecting to get out of it?
- What was your experience of the support?
- To what extent did the service(s) meet your expectations? Why? Why not?
- To what extent did the support meet your needs?
- How satisfied were you with the support you received?
- What worked well and what didn’t work so well when engaging with EWS?
- Would you use it again / recommend it to other colleagues in a similar situation?
- Any recommendations for improvement?
- Is there anything else that you want to tell us?
- Prompt: if you have been affected by the current Covid-19 pandemic, what wellbeing support services provided by the ambulance service, if any, have you tried to access?
- Prompt: how easy was it to access the service(s)?
- Prompt: what problems, if any, did you encounter when trying to access these services?
- Prompt: what has your experience been of using these services?
- Prompt: what worked well and what could be improved?
- Prompt: to what extent did they meet your expectations?
- Prompt: to what extent did the support meet your needs?
- Prompt: how satisfied were you with the support you received?
- Prompt: would you recommend it to other colleagues affected by the Covid-19 pandemic?

**Tell us about any other support or wellbeing services you have used outside of your work….**

- Which other mental wellbeing services or supports have you used when working in the ambulance service? What prompted you to seek that support?
- How easy was it to get the help you wanted?
- Possible prompts – easy to find information, timely response
- Who provided the support?
- What support were you offered?
- In what format was the support delivered?
- What were you hoping/expecting to get out of it?
- What was your experience of the support?
- To what extent did the support meet your expectations?
- To what extent did the support meet your needs?
- How satisfied were you with the support you received?
- What worked well and didn’t work so well when engaging with those services or providers?
- Any recommendations for improvement?
- Would you use it again / recommend it to other colleagues in a similar situation?
- Do you think this or something similar could be provided by your work? Would you want this? Why? Why not?
- Is there anything else that you want to tell us?
- Prompt: if you have been affected by the current Covid-19 pandemic, what other support services, if any, have you tried to access?
- Prompt: how easy was it to access the service(s)?
- Prompt: what problems, if any, did you encounter when trying to access these services?
- Prompt: what has your experience been of using these services?
- Prompt: how was the service delivered?
- Prompt: what worked well and what could be improved?
- Prompt: to what extent did they meet your expectations?
- Prompt: how satisfied were you with the support you received?
- Prompt: would you recommend it to other colleagues affected by the Covid-19 pandemic?
