# Supplementary material for: The experiences and perceptions of wellbeing provision among English ambulance services staff: a multi-method qualitative study
Source: BMC Health Serv Res. 2022 Nov 15;22:1352. doi: 10.1186/s12913-022-08729-1 (PMC9664049; doi:10.1186/s12913-022-08729-1)
Supplement: Supplementary file 4 — Appendix 4. BMC HSR paper themes and quotes. [file 12913_2022_8729_MOESM4_ESM.docx]

# Appendix 4 - BMC HSR paper themes and quotes

| **Strand of work** | **Themes** | **Quotes** |
| --- | --- | --- |
| Informant interviews | Policy development | ‘I fought for three long and hard years, with management, high up, and I mean the board, everybody, to put in place my own recruited, trained, bespoke counselling team who were experienced and trained to deal with trauma*.*’ (Trust F). |
|  |  | *‘*There was a lot of feedback from previous counsellors who would start crying, a couple of times they fainted, or they would just say, “*You must leave the service*,” none of which is what we want.’ (Trust F). |
|  | Well-being and responsibility | *‘*sometimes, there was a sense of entitlement from staff around that kind of, "You need to fix me." I think that one of the things that I recognised was we had to support people to understand that they were capable of supporting their own health and wellbeing alongside us, to provide them with the foundations, support and the ideas to look after themselves, but we couldn't fix them because that required their input into their own wellbeing.’ (Trust B). |
|  |  | ‘We cover 6,000 square miles. We’ve got over 65 ambulance stations, as well as a number of other venues that we use…Just being able to communicate with the workforce being so dispersed is the biggest challenge that I think we have. Letting them know what we’re doing, what our plans our, how they can get involved. I think that’s for me the biggest challenge.’ (Trust H). |
|  | Mental health and suicide prevention | ‘Of course, those jobs, as horrendous and emotionally demanding as they are, many of our staff, the larger majority of our staff, frankly, that’s why they joined. […] but it gives them a buzz, as opposed to going to the rubbish jobs.’ (Trust F). |
|  |  | ‘So, what they want most is for their management high up to say, “*Well done, thank you*,” not to inundate them with… well, not to pathologise something that’s normal. If we’re standing at [a burning building] and we’ve been trained to save people’s lives and we … see people burning to death, that is going to affect us, but it’s not going to destroy us unless someone says, “*Oh, my God. You must be broken. Oh, my*…” Do you see what I mean? It’s all that kind of nonsense.’ (Trust F). |
|  |  | ‘Depression is definitely a big thing, chronic stress, stress that slowly drips and accumulates, which then impacts communication, which impacts how they talk to managers, how they respond to members of the public, how they respond at home.’ (Trust F). |
|  |  | ’It’s probably worth noting that we’re putting a lot of effort into our Mental Health framework going forward”…….“I think, anecdotally, mental health is probably a big one at the moment. That can be low-level mental health. It’s not just like for PTSD or addiction forms of mental health. It could be just day-to-day stress and anxiety.’ (Trust E). |
|  |  | ‘We've got lots of ex-Forces that end up working for us who've got really significant PTSD from their experiences on tour and have spoken quite openly about historic dependence on things like alcohol. I think it's that self-medicating that people find comforting.’ (Trust B). |
|  |  | ‘we also see a lot of people with mental-health-related difficulty due to reasons outside of work. Our employee assistance programme that we commission sees two thirds more people accessing that service with personal-related problems than they are with work-related problems.’ (Trust H). |
|  |  | ‘we’re absolutely putting a huge amount of focus into mental wellbeing for our staff and our managers. And it does feel like that is absolutely the priority in terms of where our energy and time is being spent.’ (Trust H). |
|  |  | ‘I would say that over a period of four years that we have had many, many, many people with suicidal ideation. I mean, you know, daily.’ (Trust C). |
|  |  | ‘What we know we have done is stopped several people from actually completing suicide.’ (Trust C). |
|  |  | ‘We’ve got a suicide prevention task and finish group, which is starting to look at having procedures and support and guidance in place for managers who are supporting staff who are experiencing suicide ideation.’ (Trust H). |
|  | Culture of openness v stigma | ‘Culturally, it is more acceptable to come forward with mental health issue, and I think we’ve had to try and embed that internally as well, which has been particularly challenging within the ambulance service, because when I joined 13 years ago, it was very much ‘stiff upper lip and carry on.’ (Trust J). |
|  |  | ‘It is … imperative that we continue to foster an open culture of disclosure to encourage staff to seek the right support when they need it.’(Trust E policy). |
|  | Healthy lifestyle promotion and physical health | ‘Linking mental health with physical exercise, awesome. All these things are brilliant because they make sense. As a human being, if you’re chronically stressed, if you step outside and put one foot in front of the other, you are regaining control of your life.’ (Trust F). |
|  |  | ‘That’s not patronising at all, is it? No, not at all…..I love yoga and Tai Chi, but the idea that things like this will prevent someone from developing chronic stress disorder, or suicidal ideation, or, actually, taking their own lives or depression that is intense and impactful. No, it won’t. None of that [expletive] will work.’ (Trust F). |
|  |  | ‘we also do have a high level of people that have musculoskeletal conditions and injuries related to the role. Our absence level for that is pretty close to mental health, to be honest.’ (Trust H). |
|  |  | ‘our people at the moment will have to work until they’re 67, so it was interesting to see how sickness levels or people’s needs changed over that period of time in their career.’ (Trust I). |

| **Strand of work** | **Theme** | **Sub-theme** | **Quote** |
| --- | --- | --- | --- |
| Staff interview study | Supporting staff – more than lip service? | Unhealthy work | ‘No-one does. And they kind of sort of sat me down and gave me a tissue because I was crying, and he told me that I should have more confidence. And that was basically the end of it.’ Staff 3, Trust G. |
|  |  |  | ‘One shift, I went into work but I just- Every time I stood up, I nearly passed out. I had to go home. Other than that, I would’ve had to have sat at the desk for 12 hours. (Laughter) I just could not, I was going giddy and everything. I had to go home, but that counted as sickness. Even though I’d made it into work, it counted as sickness. That was one shift, I was back by the next one. A lot of them aren’t long-term for me. For others, it’s four of five weeks.’ Staff 1, Trust C. |
|  |  | Depression | ‘Yes. I mean I started off doing 111 last year because Trust G took over the 111 service for the [region Trust C] this time last year, so I’ve been in the 999 environment about 10 months now. And anything and everything that you could have experienced, I’ve heard, and as I say, it is expected, we are an emergency service, and the ambulance service receives quite a lot of calls. Well, I think the police take more than us, but the ambulance service, we do take a lot of crazy stuff. But we’re only human, we do still get personally affected by it.’ Staff 6, Trust G. |
|  |  |  | ‘I got assaulted at work about five weeks ago and the lack of support I’ve had from the Trust, I think that’s partly the reason I also signed up for it.’ Staff 3, Trust C. |
|  |  |  | ‘Yes. You know, “You’re crap, you need a new job.” You think, “Hang on a minute, you’ve called for help and all you’ve done is shout at me from the moment I picked the phone up.’ (staff 1, Trust I) |
|  |  |  | ‘…quite a lot of stress in going to patients in the sense of we didn’t really fully understand what was expected of us which was quite difficult.’ (Staff 2, Trust I). |
|  |  |  | ‘you have got no protection in there [the call centre]. You have got no screens up in-between.” Where even shops have made the effort to put a piece of Perspex up.’ ( Staff 13 Trust I). |
|  |  | Ideal v reality | ‘Yes, and it goes against the vision and values that they put in front of you and make you preach at your interview that it’s about patient care and getting best treatment at the best time. Even on jobs you’ve got them on your back, “*How long you’re going to be? You’ve been on this job whatever time*.” It’s like, “*I’m waiting to speak to a doctor*.” I’m the one that’s seen the patient. I’m the one that’s going to be stood up in front of the court.’ Staff 2, Trust G. |
|  |  |  | ‘Probably not. Looking back in hindsight, I think they probably could have offered me some more shifts with more experienced paramedics and that would have made me feel a bit more at ease.’ Staff 3, Trust G. |
|  |  |  | ‘There is an understanding there. But, with a private company who has just been giving a contract saying, “*Do this service*.” You have got to have something about you, and they didn’t. It was atrocious.’ Staff 4, Trust G. |
|  |  |  | ‘I mean I’ve had colleagues before, like when I’ve been with supervisors when they’ve had very difficult- this is just an example, this isn’t- you know, hangings or [something has 0:16:05] really affected somebody. They’ve let them go home, they’ve said, “*You need to go home*.” They’re very good at taking control and saying, “*This is actually for your mental health, not anybody else’s*.” “*I mean you’re saying that you’re okay, but I can see that you’re not. You need to go home*.”’ Staff 6, Trust G. |
|  |  |  | ‘The ones [employees] they can rely on; they don’t seem to value them. The ones that they think, “*Oh yes, she’ll turn up whatever happens*.” Staff 1, Trust C. |
|  |  |  | ‘Even the… I don’t want to say the second most important person, but the second highest in command out of the whole service came in and had a chat with us and put everyone’s mind at rest. He learned all of our names. He said that he personally… If anyone needed to have a chat, if anyone was worried about anything, we could contact him. It was so lovely that someone so high up, with so much responsibility and so many other things to worry about, was so caring and so considerate to the way we were feeling. That was just really, really, lovely, so I thought I’d mention that.’ Staff 5, Trust G. |
|  |  |  | ‘Oh, definitely, yes. I don’t know. There is just a happier vibe. We have got the director of the 111 service in the [area]. He comes round every weekend and he says hello to every member of staff.’ Staff 4, Trust G. |
|  |  | Engagement with the health and well-being agenda | ‘I personally think that people do need to take a lot of responsibility for their own wellbeing.’ Staff 3, Trust C. |
|  |  |  | ‘They expect you to say, “*Oh yes, I’ll go in*.” The ones that go off all the while, it seems to me that they get rewarded. They get all the support they need. One particular guy, since he passed his assessment, he’s not done a straight 6- He’s been off, I would say… He’s been a year, 14 months, I would say he’s probably been off 8 months. Admittedly, his brother… He had a death in the family with his brother, so that knocked him back again. He’d been off before that, previously, so… He’s been getting all the support. You think, “That lady there, that’s been sat there for the last 12 years, that really needs some help and…” They just expect it of the ones that turn up day in and day out.’ Staff 1, Trust C. |
|  | Variable experiences of health and well-being services |  | ‘Yes. When I was at an all-time low and struggling they left me two weeks, so they’re not going to be bothered about someone that’s just worried because of their job.’  Staff 2, Trust G. |
|  |  |  | ‘They always listen to you. You can always hear them making notes and stuff, to make sure they don’t forget anything if they talk to you again. To be honest, the lady I speak to, I don’t think she really needs to take many notes. We’re quite familiar with each other now, and you build up a bit of a relationship with them. When you need to access things, it’s always there. They’ll always be someone, in one of the services, who will answer the phone or get back to you as quick as they can.’ Staff 1, Trust G. |
|  |  |  | ‘When I was going through my divorce I worked for a different service then. They were supportive and they put in obviously referrals to see a counsellor but my GP also put in a service as well. It came through at the same time but I just used the Ambulance Service one because obviously it was better using the work one. I found that quite helpful and got a lot of things off my chest that I suppose you don’t realise bothers you until you start talking about things.’ Staff 2, Trust G. |
|  |  |  | ‘I know the NHS service is absolutely inundated. I've just been discharged unfortunately because of the coronavirus and they're not doing face-to-face. The woman that I was seeing was leaving, so they've discharged me without completing the course. Given me some online stuff to do.’ (Staff 4. Trust I). |
|  |  |  | ‘They’ve got the TRiM where if you feel that you need to talk to somebody about a certain call, they’ll listen to the call and be able to give you their opinion and give you some sort of counselling towards if it was a particularly bad one.’ (Staff 1) |
|  |  |  | ‘Then they can come back and say, “I need TRiM.” It seems to work for them. However, for others, they go off for months because of it. Not because of TRiM but because it’s not worked’ (Staff 1, Trust I). |
|  |  |  | ‘They’ve got the TRiM where if you feel that you need to talk to somebody about a certain call, they’ll listen to the call and be able to give you their opinion and give you some sort of counselling towards if it was a particularly bad one.’ Staff 1, Trust C. |
|  |  |  | ‘we will be automatically referred, but equally, one person's nasty job may not be another's.’ (Staff 7, Trust I). |
|  |  |  | ‘TRiM […] I had several referrals to them over the years and never heard from them.’ (09) |
|  |  | Culture of openness v stigma | ‘Oh, definitely, yes. I don’t know. There is just a happier vibe. We have got the director of the 111 service in the [region Trust G]. He comes round every weekend and he says hello to every member of staff.’ Staff 4, Trust G. |
|  |  |  | ‘Yes. The support, from management, is nowhere near as good as the support you get outside. They, very much, just want you to be doing your job. They don’t want you to be off work. They just want you to be there doing it. They don’t want to be paying you if you’re not there, things like that. The other support you get is brilliant. Even with management… I think it happens most places, doesn’t it, management just sort of want you to man up a bit, get on with it, if that’s the right thing to- Well I did get told that once by management, to be honest, “*Just man up*.” That didn’t go down very well.’ Staff 1, Trust G. |
|  | Relationships between staff and their employer | Mutualism | ‘My manager, particularly, is so lovely, he is so helpful. I’ve got a few health problems. Whenever I’ve got a problem, I have to take some time off. He’s the first person to come and ask me if I’m okay and have a chat with me about what was wrong and, “*What are you doing to*…? *Have you spoken to someone*?”  Staff 5, Trust G. |
|  |  |  | ‘They helped me with juggling some hours, start times and… I’ve said that I think I can’t do 12 hour nights or I can’t do 6:00 to 6:00, 18:00 to 06:00 anymore. They said, “*Come up with what you want to do, then we’ll see if we can fit it in*?” I don’t like to think about dropping it but I think, for myself, I’ve got to because I’m just not sleeping. If can get home by 4:00 o’clock in the morning- They’ve said they’ll look at it.’ Staff 1, Trust C. |
|  |  |  | ‘I’ve also got a colleague that’s been fighting just to reduce her hours for a few hours. She’s been fighting for it for three years. It’s just tipped her over- She lost her husband. As soon as she got back to work, they expected her to be back at work and normal. It just didn’t happen. She’s had no support, really, whatsoever. She’s now off sick.’ (Staff 1, Trust I). |
|  |  |  | ‘It makes you feel like, mentally, the Trust is trying to give you back something so you’re getting something out of the Trust rather than them just constantly draining the life out of you.’ (Staff 2, Trust I). |
|  |  | Presenteeism | ‘It’s taken all my will power not to go in this weekend….I’m on antibiotics, now, for an infection…..it’s not anything major but I- I wanted to go in this weekend because I know they’re going to be busy and it’s my team, but I had to think of myself. I’m really only back to normal the last 6 months, after 18 months, so I can’t afford- As much as I want to go in, I can’t afford to risk it really at the minute.’  Staff 1, Trust C. |
|  |  |  | ‘you're sitting at home feeling a bit of a fraud really, because I'm getting paid to sit at home, you know?’ (Staff 8. Trust I). |
|  | Resilience or resignation | Decline and deterioration | ‘We work such awful hours. There are loads of studies that show our life expectancy and our health is reduced over the long term because of the type of work we do.’ Staff 2, Trust C. |
|  |  |  | ‘Now I’m getting older and getting a bit creakier…I needed to get myself fit in several ways – general stamina, but also upper body strength.’ Staff 16, Trust C. |
|  | Coping strategies for stress and ill-health |  | ‘I do zone out a little bit when things get a bit too much.’ Staff 4, Trust C. |
|  |  |  | ‘I find that a nice release to go and exercise.’ Staff 3, Trust C. |
|  |  |  | ‘I have a five-minute kip and I am right as rain afterwards.’ Staff 14, Trust C. |
|  |  |  | ‘You crack a joke and it doesn't always work, so you try something else to alleviate the situation. But yes, humour is definitely a coping mechanism, without a shadow of a doubt, yes. Definitely.’ Staff 2, Trust I. |
|  |  |  | ‘Yes. It seems very, very, backwards but… I don’t know why, it’s just we’re quite good at detaching from it. Everyone checks on everyone else and makes sure everyone is okay.’ Staff 5, Trust G. |
|  |  |  | ‘I think the CBT has helped me more so, because that gives you the tools […] rather than - Counselling doesn't really give you that.’ Staff 4, Trust C. |
|  |  |  |  |
|  |  |  |  |
|  |  |  |  |
|  |  |  |  |
|  |  |  |  |
|  |  |  |  |
|  |  |  |  |
|  |  |  |  |
|  |  |  |  |
|  |  |  |  |
|  |  |  |  |
|  |  |  |  |
|  |  |  |  |
|  |  |  |  |
|  |  |  |  |
|  |  |  |  |
|  |  |  |  |
|  |  |  |  |
|  |  |  |  |
|  |  |  |  |
|  |  |  |  |
|  |  |  |  |
|  |  |  |  |
|  |  |  |  |
|  |  |  |  |
|  |  |  |  |
|  |  |  |  |
|  |  |  |  |
|  |  |  |  |
|  |  |  |  |
